# Understanding the psychology of Filipino athletes through emotional intelligence and conflict behaviors

**DOI:** 10.3389/fpsyg.2026.1716594

**Published:** 2026-04-23

**Authors:** Qunzhen Su, Junhui Tan, Kris G. Lobo, Bethzaida Mary Joy Dela Cruz

**Affiliations:** 1 College of Physical Education, Hunan University of Technology, Zhuzhou, Hunan, China; 2 School of Physical Education, Guangxi Minzu Normal University, Chongzuo, Guangxi, China; 3 Department of Human and Family Development Studies, University of the Philippines Los Banos, Los Banos, Laguna, Philippines; 4 Office of Expanded Tertiary Education Equivalency and Accreditation Program, Lyceum of the Philippines University, Manila, Philippines

**Keywords:** conflict behavior of athletes, emotional intelligence of athletes, Filipino athletes, Filipino Psychology, Filipino values

## Abstract

This study examines the levels of emotional intelligence (EI) and conflict behavior (CB) among Filipino university athletes (*N* = 208) and explores differences across sex, sport type, and sport category. This study employed an exploratory cross-sectional quantitative research design, appropriate for contexts where empirical evidence of the constructs of interests remains limited and the aim is to identify patters of association. Data were collected via the Wong and Law Emotional Intelligence Scale (WLEIS) and the Dutch Test for Conflict Handling (DUTCH). Given the ordinal nature of the data and non-normal score distributions, non-parametric statistical analyses were employed, specifically Mann–Whitney U test for two group comparisons and the Jonckheere-Terpstra test for ordered comparisons across sports categories. Results indicate that Filipino athletes generally exhibit high EI levels (*md* = 6). Significant differences in EI were found by sex (e.g., items related to self-emotion appraisal, other’s emotion appraisal, regulation of emotion) and sport type (e.g., an item related to other’s emotion appraisal). Conflict behavior differences were observed by sex (e.g., conflict behaviors such as accommodating, forcing, avoiding) but not by sport type or event category. Findings are interpreted through the lens of Filipino Psychology, specifically the kapwa framework of insider (Hindi Ibang Tao) and outsider (Ibang Tao) relational categories, providing a culturally grounded understanding of emotional and conflict processes in Filipino sports contexts. The EI and CB of Filipino athletes can be best understood through *loob* (inner self), *pakiramdam* (feeling for another), *hiya* (modesty), *pakikisama* (getting along with others), *pakikipagkapwa-tao* (humanity towards others), pag*-iwas gulo* (avoiding trouble; maintaining harmony). Implications for EI training and culturally responsive conflict management interventions are discussed.

## Introduction

Emotional intelligence constitutes a multifaceted constructs encompassing the capacity to perceive, utilize, comprehend, manage and regulate emotions, both within oneself and in others, which plays pivotal role in navigating social complexities and achieving adaptive outcomes (Mancini et al., 2022; Lobo, 2023). Athletes who cultivate high levels of emotional intelligence frequently exhibit enhanced performance, resilience, and interpersonal relationship in the realm of competitive sports (Kopp and Jekauc, 2018). As noted by Murphy (2009) the ability to accurately assess and express emotions in oneself and others is a cornerstone of emotional intelligence that enables to understand their own emotional states and those of their teammates and opponents. This awareness allows for more effective communication, collaboration, and conflict resolution, fostering a positive and supportive team environment (Liu et al., 2025).

In the Philippines, sports are integral aspect of life for Filipinos. It is embedded in the culture and values and can be also seen in the national identity. More specifically, Filipino athletes have gained recognition in international competitions in terms of basketball, boxing, weightlifting, and volleyball. This is because, on one hand, Filipino sports psychologists contribute significantly by helping athletes and coaches achieve their goals through programs designed to improve motivation, confidence, and concentration. On the other hand, coaches and trainers also play an important role in assisting athletes to acquire the necessary knowledge and skills to reach optimal performance, while also emphasizing the importance of ethical behavior, teamwork, and sportsmanship. They ensured that athletes are well-rounded individuals, and that their well-being is prioritized (Nozaki and Koyasu, 2013; Gunkel et al., 2013).

While this is the case, when it comes to the role of emotional intelligence (Kopp and Jekauc, 2018) on conflict management further empirical investigation is needed, especially in countries like the Philippines with a cohesive emphasis on group (Boncompagni and Casagrande, 2019; Liu et al., 2025; Ding et al., 2024; Lobo, 2023). It is imperative to determine the relationship between EI and conflict resolution in the context of sports (Ros-Morente et al., 2022). The pressure on athletes may result in a team member attributing blame to another or even the coach when outcomes deviate from expectations; if one is not careful, it could result in a toxic team environment. In this context, we refer to EI as a psychological resource because the ability to understand one’s emotion and regulate emotions in these tensed situations is key for one to not be influenced by negative emotions that emanates from tension and disagreements.

Considering the wider literature, we found four gaps that we are compelled to address. First, the implementation of EI training programs in sports remains limited (Ros-Morente et al., 2022) particularly in the Philippines as there were only few published article related the influence of EI (Santiago and Mateo, 2020; Lobo et al., 2022). Many coaches and sports organizations may not fully understand the benefits of EI or may lack the resources and baseline data on it as well as expertise to effectively integrate EI training into their existing programs. This is also consistent with the study of Diotaiuti et al. (2021) that coaches has influence on how athletes perceive and demonstrate competence, performance and endurance in sports. In this vein, a necessity for enhanced awareness and education about the significance of EI in sports, alongside the creation of evidence-based emotional intelligence’s training programs tailored to the specific needs of athletes (O’Connor et al., 2019).

Second, the ability to harness emotions to facilitate cognitive processes is another crucial aspect of emotional intelligence, enabling athletes to leverage their emotional states to enhance focus, creativity, resilience, and problem-solving skills. For example, athletes can use positive emotions like excitement and enthusiasm to fuel their performance, while also regulating negative emotions like anxiety and frustration to maintain composure under pressure (Wong and Law, 2002). As noted by Lobo (2023), this aspect was not thoroughly explored in the literature.

Third, EI is also essential in addressing conflict as it equips individuals with the skills to resolve disputes constructively, which is particularly valuable in sports settings where disagreements between teammates or coaches can arise (Shih and Susanto, 2010). More than that, it also influences what kind of conflict behavior someone would use to deescalate or escalate the tension. For instance, athletes with high EI may be are more likely to employ collaborative conflict resolution strategies, seeking mutually beneficial solutions that preserve relationships and promote team cohesion (Burcea and Sabie, 2020; Jenita et al., 2024). However, to fully understand this, empirical studies are needed to explore the precise mechanisms through which EI impacts conflict resolution in sports teams.

Lastly, the role of EI on athletes can be best understood through the cultural context. Cultural values and norms can shape individuals’ emotional expression, interpretation, and regulation, thereby influencing the manifestation and impact of EI in sports settings (Kopp et al., 2021). For instance, cultures that emphasize collectivism and group harmony may prioritize emotional regulation and empathy to maintain team cohesion, while cultures that value individualism and assertiveness may encourage athletes to express their emotions more openly (Mitić et al., 2020; Mon-López et al., 2023). Athletes seek team support when confronted with different stressors, acknowledge that their teammates’ emotions affect them, attempt to control their own emotions to not disturb teammates, and also try to control teammates’ emotions to enhance team performance (Mitić et al., 2020). In the Philippines, the concept of outsider and insider is a significant factor influencing interpersonal dynamics, where individuals tend to form strong bonds within their in-group and maintain a more reserved stance towards those perceived as outsiders. This dynamic significantly impacts emotional expression and regulation within sports teams, as athletes may exhibit different emotional responses and conflict resolution strategies depending on whether they perceive others as part of their inner circle or as external to it. This cultural lens is critical for understanding how emotional intelligence manifests in team cohesion and conflict resolution among Filipino athletes, necessitating research that integrates these specific socio-cultural elements into its analytical framework such as the Filipino Psychology of developed by Enriquez (1976, 1977, 1978). To date, the understanding of this sphere is underexplored and further cross-cultural research is needed to examine how cultural factors moderate the relationship between EI and conflict behaviors, as well as to develop culturally sensitive EI interventions that resonate with athletes from diverse backgrounds (Peris-Delcampo et al., 2024).

Based on this, we are motivated by the study of Kopp et al. (2021), researchers who claimed that, EI is a key for improving sports performance which highlights the importance of understanding and managing emotions in competitive environments. As such, EI appears to be an essential component of success in athletic sports, optimizing sports performance.

In line with this, since the relationship between EI and conflict management still remains relatively unexplored within sports psychology research, we are also compelled to conduct an exploratory study to investigate their correlates with demographic antecedents (Shih and Susanto, 2010). With this in mind, our overall research question is: “How does EI of Filipino athletes influence conflict management*”*. To address this, we pose the following objectives:

*RQ1. What are the levels of EI and conflict behavior scores of Filipino athletes?*

*RQ2. Do Filipino athletes differ significantly in their emotional intelligence scores across sex?*

*RQ3. Do Filipino athletes differ significantly in their emotional intelligence scores across sport type and sport category (individual vs. team)?*

*RQ4. Do Filipino athletes differ significantly in their conflict behavior scores across sex, sport type, and sport category?*

*RQ5. How can the significant patterns observed in EI and conflict behavior be interpreted through the lens of Filipino Psychology, specifically the kapwa framework of insider (Hindi Ibang Tao) and outsider (Ibang Tao) relational categories?*

In this vein, it is important to note that this study operates on two distinct levels of inquiry. The first level, addressed by RQ1 through RQ4, is empirical: it involves the quantitative measurement of EI and conflict behavior and the statistical testing of group differences across sex, sport type, and sport category using non-parametric analyses. The second level, addressed by RQ5, is interpretive and conceptual: it draws on Filipino Psychology, specifically Enriquez (1977) kapwa framework, as a culturally grounded lens through which to contextualize and make sense of the empirical patterns identified. The Filipino Psychology framework was not operationalized as a measured variable in this study, nor was it subjected to statistical testing. Rather, it functions as a theoretical scaffold for culturally situated interpretation. We make this distinction explicit to ensure transparency about the epistemological scope of our claims and to avoid conflating statistical inference with cultural theorization.

By doing so, we hope to advance the theoretical understanding of EI in sports settings and provide evidence-based recommendations for sports organizations seeking to enhance athlete performance and well-being through EI training and development. Aside from this, future studies should consider examining the role of EI in predicting athletic success, leadership emergence, and injury recovery, as well as exploring the potential for using technology-based interventions to deliver EI training to athletes (Wong and Law, 2002). Ultimately, this research will advance the literature of EI in relation with sports development.

### Theoretical framework on emotional intelligence and athletic performance

**Figure 1:**
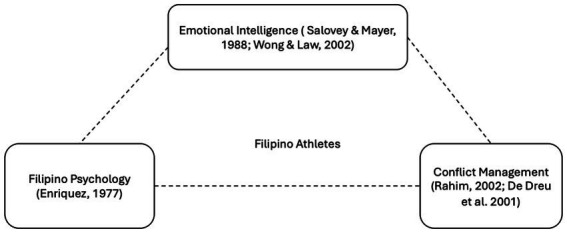
Theoretical framework. Source: authors.

Figure 1 illustrates Emotional Intelligence Theory, Conflict Management Theory and Filipino Psychology. The framework proposes that these interrelated constructs collectively facilitate the development of Filipino athletes’ sports performance through EI and conflict management.

*Emotional intelligence theory.* The core theoretical framework of this study is based on the ability-based model of emotional intelligence. Coined by Salovey and Mayer (1990), EI is the ability of an individual to monitor one’s and others’ feelings and emotions, to discriminate among them, and use this information to guide one’s thinking and actions (Wong and Law, 2002). This model suggests that EI encompasses the capacity to perceive, understand, manage, and utilize emotions effectively (O’Boyle et al., 2010). Following the definition of Wong and Law (2002) and Shih and Susanto (2010), perceiving emotions involves accurately recognizing and identifying emotions in oneself and others. Understanding emotions entails comprehending the meaning of emotions and how they evolve over time. Managing emotions refers to the ability to regulate and control one’s own emotions, as well as influence the emotions of others (Mayer et al., 2016). Utilising emotions involves leveraging emotions to facilitate cognitive processes, such as problem-solving, decision-making, and creativity (McGinnis, 2018). In other words, the ability-based model emphasizes that EI is personality trait and a set of cognitive abilities that can be developed and improved through training and practice (Corbí et al., 2019).

*Conflict management theory.* The conflict management theory suggests that conflict is a natural and inevitable part of human interaction, and effective conflict management is essential for maintaining positive relationships and achieving shared goals. In this study, we used Rahim’s integrative model of conflict management styles^1^. According to Rahim (2002), conflict management styles are classified based on two dimensions which are concern for self and concern for others. Concern for self refers to the degree to which an individual prioritizes their own needs and interests during conflict, while concern for others reflects the extent to which an individual considers the needs and interests of others. To measure these styles, there are five conflict behaviors notably integrating, obliging, dominating, avoiding, and compromising. Integrating involves a high concern for both self and others, where individuals seek to find mutually beneficial solutions that satisfy the needs of all parties involved. Obliging entails a low concern for self and a high concern for others, where individuals prioritize the needs of others and may sacrifice their own interests to maintain harmony. Dominating involves a high concern for self and a low concern for others, where individuals prioritize their own needs and may use power or authority to impose their will on others. Avoiding entails a low concern for both self and others, where individuals withdraw from conflict or avoid addressing the issues altogether (Assi et al., 2022). Compromising involves a moderate concern for both self and others, where individuals seek to find a middle ground or compromise that partially satisfies the needs of all parties involved (Messarra et al., 2016). Rahim’s theory has been used as a basis for research in conflict management (Havenga and Visagie, 2006).

*Filipino Psychology.* In this study, the researchers will also use a guiding framework which is the Filipino Psychology theory to better understand the utilization of EI and conflict behavior styles of Filipino athletes. Filipino Psychology, or *Sikolohiyang Pilipino* coined by Enriquez (1976), emphasizes the importance of understanding the local context and culture when studying human behavior in the Philippines. *It* is anchored to the concept of “kapwa” which means shared identity. Kapwa has two categories: Ibang-Tao (outsider) and Hindi Ibang-Tao (one of us). Ibang-Tao (“outsider”) is further classified into five interaction levels: pakikitungo (courtesy), pakikisalamuha (mixing), pakikilahok (joining), pakikibagay (conforming), and pakikisama (being united with). Hindi Ibang-Tao is further classified into three interaction levels: pakikipagpalagayang-loob, pakikipag-kaisa, and pakikipagka-isa. Pakikitungo means courtesy which means dealing with other people in accordance with proper behavior; pakikisalamuha means act of mixing which means mixing with other people; pakikilahok means act of joining which means participating or joining other people in their activities; pakikibagay means conformity which means being in conformity with the majority; pakikisama means being united with the group. Pakikipagpalagayang-loob means act of mutual trust; pakikipag-kaisa means being one with others; pakikipagka-isa means being united with others. Understanding Filipino values and cultural norms, such as collectivism, respect for authority, and the importance of maintaining harmonious relationships, can provide valuable insights into how Filipino athletes perceive and respond to conflict situations and how they use their emotional intelligence.

### Novelty of the study

#### Integrating emotional intelligence, conflict management, and Filipino Psychology in understanding Filipino athletes

This study proposes a conceptual model below that integrates emotional intelligence theory, conflict management theory, and Filipino Psychology to deepen our understanding of the Filipino athlete’s psychological functioning when it comes to tension and disagreements. Emotional Intelligence (EI), based on the ability-based model by Salovey and Mayer (1990), provides insight into how athletes perceive, regulate, and utilize emotions in managing both internal states and social interactions. In the high-pressure context of sports, these emotional skills are essential in maintaining focus, resilience, and empathy among team members. Complementing this, Rahim’s Conflict Management Theory introduces five distinct styles, such as integrating, obliging, dominating, avoiding, and compromising, each representing varying levels of concern for self and concern for others. These styles explain how athletes manage interpersonal tensions and maintain relationships within their teams. To contextualize these psychological processes within the Filipino experience, the study draws on Filipino Psychology or Sikolohiyang Pilipino, particularly the cultural value of kapwa (shared identity). Through interaction levels such as pakikisama (being united with the group) and pakikipagpalagayang-loob (mutual trust), Filipino athletes demonstrate culturally rooted ways of managing emotions and conflict.

Taking this integrated approach, the research aims to uncover the unique interplay between emotional competencies, conflict resolution strategies, and indigenous cultural values in shaping the performance and well-being of Filipino athletes (Lobo et al., 2022).

For instance, in relation to EI, the self-oriented dimensions—SEA and ROE—are closely tied to Filipino cultural values of *loob* (inner self), *hiya* (propriety), and *amor propio* (self-respect). The ability to recognise and regulate one’s emotions is central to maintaining dignity and avoiding shame in social interactions. This is particularly crucial in *Ibang Tao* contexts, where formal distance requires emotional restraint and self-control.

In line with this, the other-oriented dimensions—OEA and UOE—reflect relational competencies consistent with *pakikiramdam* (shared inner perception or sensitivity) and *pakikipagkapwa* (fellowship). These dimensions emphasise attentiveness to the emotions of others and the capacity to mobilise emotions to foster solidarity and cooperation. They are most salient in *Hindi Ibang Tao* contexts, where openness and trust allow for genuine emotional expression and collaborative engagement.

In addition, when it comes to conflict management, self-oriented styles such as forcing and avoiding reflect self-preservation. Forcing, characterised by assertive pursuit of one’s goals, may be legitimised in hierarchical or competitive situations, often seen in *Ibang Tao* contexts where distance and authority are respected. Avoiding, on the other hand, reflects the cultural value of *hiya* and the Filipino tendency to sidestep open confrontation in order to maintain harmony. Other-oriented styles—Yielding, Compromising, and Problem Solving—are more consistent with *pakikibagay* (adjustment), *pakikisama* (companionship), and *pakikiisa* (oneness). Yielding involves deference to others in order to preserve smooth interpersonal relations, while Compromising represents a balanced adjustment to competing needs. Problem Solving, as a cooperative and integrative style, resonates most strongly with *Hindi Ibang Tao* relationships, where trust and openness enable collaborative resolution.

We acknowledge that the integrated theoretical framework presented here is broader in scope than what a single cross-sectional study can empirically verify. The framework is intended to serve two functions: first, to situate the empirical findings within a coherent theoretical context; and second, to provide a roadmap for future research that may test the proposed relationships among Filipino Psychology, emotional intelligence, and conflict behavior using more methodologically comprehensive designs, including sequential mixed-methods, longitudinal, or culturally validated psychometric approaches.

#### Conceptual model

Considering the experience of a Filipino athlete competing in a high-pressure environment, in such settings, success is not determined solely by physical skill or tactical discipline, but also by the athlete’s ability to navigate complex emotional and social dynamics within a team. This framework situates Filipino athletes’ performance within a culturally rooted understanding of behavior, highlighting the importance of kapwa, or shared identity, as conceptualised in Enriquez’s (1977) Filipino Psychology. Athletes interact with others based on relational categories, Ibang-Tao (outsiders) and Hindi Ibang-Tao (one of us), which guide behavior through values such as pakikibagay (conformity), pakikisama (maintaining harmony), and pakikipagka-isa (unity). These social norms shape how Filipino athletes perceive group membership, establish trust, and maintain cohesion.

At the same time, the ability to manage emotions plays a crucial role. Drawing from the ability-based model of Emotional Intelligence (Salovey and Mayer, 1990; Wong and Law, 2002), the framework identifies key emotional competencies such as self-emotion appraisal, others’ emotion appraisal, and the use of emotion. These competencies help athletes remain focused, empathetic, and responsive during performance and interpersonal interaction. When disagreements or tensions arise, athletes rely on culturally influenced conflict management strategies such as accommodating, avoiding, problem-solving, and compromising as described by De Dreu et al. (2001). These responses are not only shaped by individual temperament but also by emotional awareness and the cultural imperative to maintain relational harmony.

Furthermore, the insider–outsider distinction of *kapwa* provides the relational conditions that determine which emotional intelligence (EI) competencies are activated and which conflict strategies are used. When individuals interact with *Ibang Tao* (outsiders), self-focused EI dimensions such as Self-Emotion Appraisal (SEA) and Regulation of Emotion (ROE) become more salient. These competencies help individuals regulate their feelings and maintain propriety, which in turn prevents situations that may provoke *hiya* or social embarrassment. In such contexts, conflict management often takes the form of self-oriented strategies like Avoiding or Yielding. These approaches allow individuals to minimize direct confrontation, preserve face, and protect social harmony in formal or distant relationships. By contrast, interactions with *Hindi Ibang Tao* (insiders) bring other-focused EI dimensions to the forefront. Others’ Emotion Appraisal (OEA) and Use of Emotion (UOE) enable relational sensitivity and emotional responsiveness, allowing individuals to recognize and attune to the emotions of others. These competencies foster cooperative conflict management strategies such as Compromising and Problem Solving, which are rooted in empathy, mutual trust, and shared understanding. In insider relationships, emotional openness is not only acceptable but expected, making it possible to engage in constructive collaboration that strengthens bonds of solidarity.

**Figure 2:**
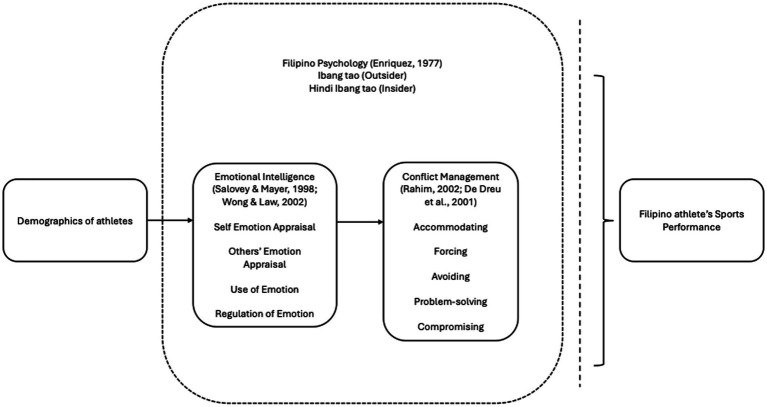
Conceptual framework of the study. Source: authors.

Together, these elements of the conceptual framework in the Figure 2, notably Filipino positionalities (outsider, insider), emotional intelligence, and conflict management, interact within the broader context of an athlete’s demographic background and team environment. This integrated framework below provides a comprehensive lens through which to understand the emotional, cultural, and eventually behavioral dimensions of Filipino athletes’ sports performance.^2^

### Hypothesis of the study

Considerable research was devoted to understanding the positive association of EI with performance, leadership, education, negotiation and decision making. However, to our knowledge, little attention was dedicated to the understanding of the EI relationship with conflict behavior in the context of Filipino athletes or sports in general. This is because most of studies on EI were directed towards understanding the context of performance in organizational research particularly in the Philippine context (Lobo, 2023; Santiago and Mateo, 2020). This means that EI and CM influence how athletes self-motivation affects their performance and their ability to solve tensions and disagreements with their fellow athletes influence their performance. Based on these previous studies, we hypothesize that the distribution of the dependent variable is the same as the groups (two or more) or equivalently the two groups has equal medians. This means that:

1 For sex (male vs. female) and categories (individual vs. team-based) comparisons:

*Ho: There is no significant difference among sex and categories, and emotional intelligence or conflict behavior.*

*Ha: There is a significant difference among sex and categories and emotional intelligence or conflict behavior.*

2 For sports category comparisons:

*Ho: There is no ordered difference between different sports categories and emotional intelligence or conflict behavior.*

*Ha: There is an ordered difference between different sports categories and emotional intelligence or conflict behavior.*

## Method

### Research design

**Figure 3:**
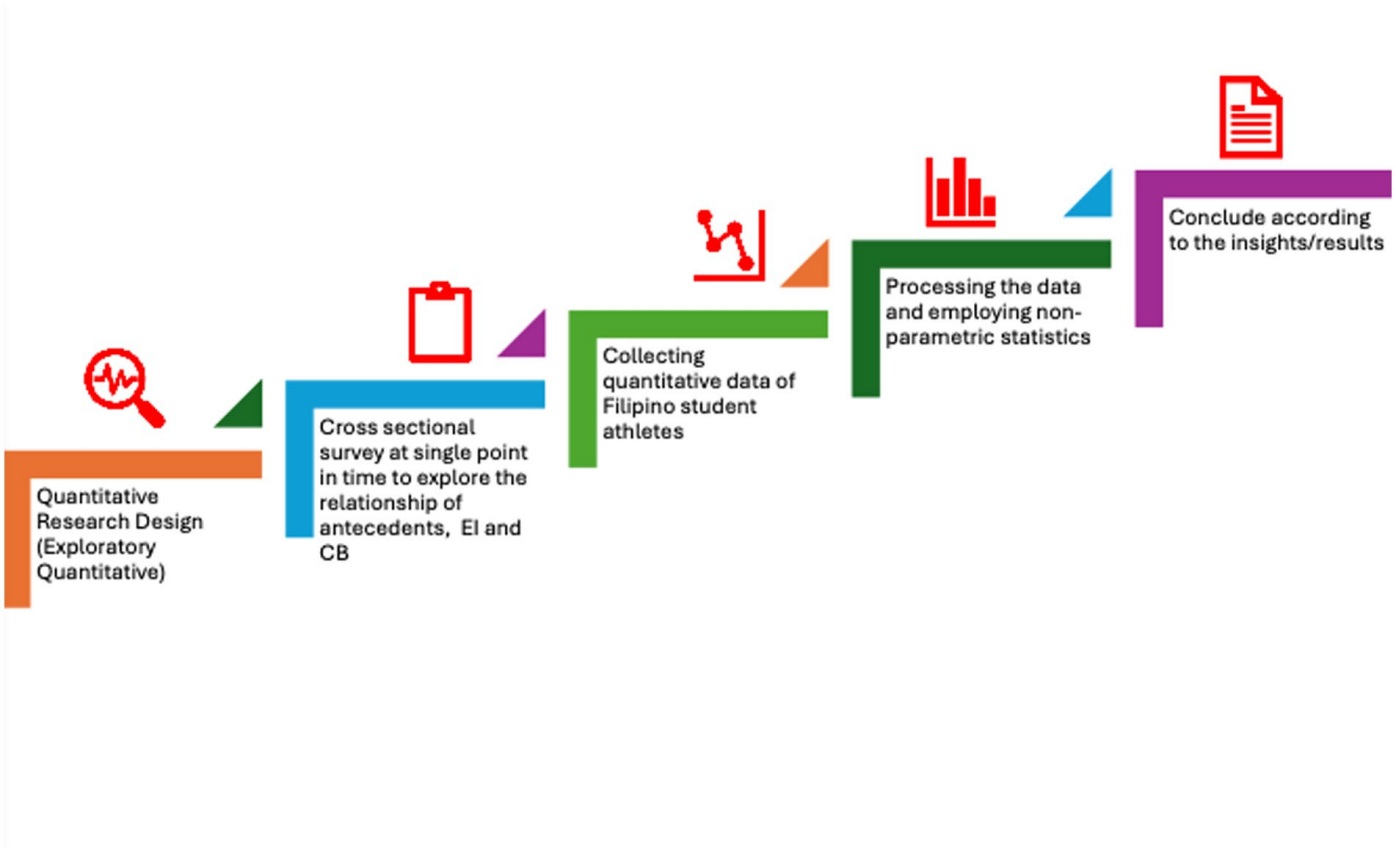
Research approach. Source: authors.

This study employed an exploratory quantitative design using cross-sectional data to examine the relationship between emotional intelligence and conflict management among Filipino university athletes (please refer to the Figure 3). An exploratory quantitative approach was appropriate because empirical research on these constructs within the Philippine sports context remains limited and the study’s objective sought to identify initial patterns, differences and associations, and potential theoretical linkages. It allows us to measure variables objectively, without manipulations, and to determine meaningful relationships that need deeper investigations. Furthermore, the use of cross-sectional data also enabled us to assess EI and conflict behavior at a single point in time wherein we capture athlete’s responses within their natural training without employing experimental manipulation. This design also accommodates the constraints of athletic schedules and reduces participant burden as well as making it more suitable for data collection within the university sports settings. Aside from this, this design is appropriate given the study’s focus on describing levels of emotional intelligence and conflict management styles, and on testing whether significant relationships exist between these constructs (Kopp and Jekauc, 2018).

### Research participants

The participants of this study were Filipino athletes in the university who were actively engaged in competitive sports at the time of data collection.

In terms of inclusion criteria, participants were required to meet all of the following criteria to be included in the study:

(a) Must be at least 18 years of age at the time of data collection.
(b) Must be currently enrolled in a university and formally affiliated with a sports team, varsity programme, or recognised sports club.
(c) Must have participated in at least one formal inter-institutional or intramural competition within the 12 months preceding data collection.
(d) Must be able to read and understand English, as the instruments were administered in English without translation.

In terms of exclusion criteria, participants were excluded if they met any of the following conditions:

(a) Submitted incomplete questionnaires, defined as missing responses on more than 10% of items in either instrument.
(b) Demonstrated straight-lining behavior or invariant response patterns across all items, identified during data cleaning.
(c) Were not currently active in any sport at the time of data collection, such as those on medical leave or who had formally withdrawn from their sports programme.

These criteria were applied during the data cleaning phase, which resulted in the final valid sample of 208 participants from an initial pool of respondents.

Furthermore, athletes from both individual and team sports were also considered to ensure a wide representation of sports experiences. It is worthy to note that the survey did not pre-select specific types of sports. Instead participation was open to all university athletes who met the inclusion criteria. As a result, athletes from a range of individual and team sports were represented in the sample including table tennis, swimming, chess, badminton, basketball and volleyball. In addition, participants from drum squad or pep squad were also included as this group is formally recognized within Philippine universities as part of athletic competition.

### Research procedure

In terms of research procedure, it was carried out in a systematic and ethically guided manner to ensure the integrity of the data collection process and the protection of participant rights. All steps were conducted in accordance with established standards for behavioral research involving human participants. It was designed to be consistent both online and face-to-face modes of administration. Researchers made sure that all participants received same instructions, instruments and level of guidance regardless of how they were reached. The following steps outline the process in sequential order.

Step 1—Institutional Approval. Prior to any data collection activity, the research team submitted an application to the university’s Institutional Review Board (IRB). Ethical clearance was granted before recruitment commenced. Permission was also sought from the relevant university sports offices, coaches, and varsity coordinators at each participating institution. In addition, referrals (using snowball method) were also utilized which was essential in reaching athletes who were not accisble through formal institutional channels alone and helped improve the overall coverage and diversity of the sample.

Step 2—Participant Recruitment. Initial contact was made through formal written requests to coaches and sports administrators. Eligible athletes were then informed about the study by their respective coaches or coordinators, who distributed study information sheets.

Step 3—Informed Consent. All eligible athletes were provided with an informed consent form prior to data collection. The form described the purpose of the study, the voluntary nature of participation, confidentiality provisions, and the right to withdraw at any time without consequence. No data were collected from any participant who had not provided written or confirmed digital consent.

Step 4—Survey Administration. Data collection was conducted through two modes, chosen to accommodate the varying schedules and connectivity of athletes across regions. The first mode was an online survey administered via a secure link, distributed through email and messaging platforms with the assistance of coaches. The second mode was a face-to-face paper-based survey conducted after training sessions or competitions at accessible venues. Researchers were present during face-to-face sessions to clarify instructions and ensure consistent administration. Both modes used identical question wording, response scales, and section ordering to maintain procedural equivalence.

Step 5—Data Collection Period and Geographic Coverage. Data were collected across multiple rounds due to an initially insufficient sample size. The expanded data collection included athletes from Luzon, Visayas, and Mindanao, representing different regions of the Philippines. The use of both online and face-to-face modes was intended to improve accessibility and minimise non-response among athletes with limited internet access.

Step 6—Data Cleaning and Screening. Upon completion of data collection, all responses were encoded in SPSS. Screening procedures included checks for missing data, duplicate entries, outliers, and invariant response patterns. Responses that did not meet the inclusion criteria or that exhibited problematic response patterns were excluded. Following this process, 208 responses were retained as valid for analysis.

Step 7—Statistical Analysis. Cleaned data were analysed using SPSS. Descriptive statistics were computed first, followed by checking statistical violations then implementing inferential analyses as described in the Statistical Treatment section.

### Sampling

A convenience sampling technique was employed. It is a non-probability sampling technique commonly used in behavioral and sports research where access to participants is shaped by organizational schedules, training routines and institutional gatekeeping. We chose this approach because Filipino university athletes often have strict training timetables and limited availability. It allowed us to reach athletes who were accessible during the data collection period while still ensuring representation across various sports. In line with this, we acknowledge that although convenience sampling does not provide full representation, it is appropriate for an exploratory research that aims to understand patterns of EI and conflict behavior within this specific group.

The participants were recruited through coaches, sports coordinators, and university varsity programmes and clubs. Initial contact was made by requesting permission from the university and its respective coaches. Recruitment was conducted in two modes: online recruitment and referral employing online and face-to-face coordination. This approach helped maximize engagement and reduce non-response specifically among athletes with limited access to online platforms.

The final sample included athletes from different regions of the Philippines (Luzon, Visayas, and Mindanao). The study was initially launched with a survey, however, the first round of data collection did not yield an adequate sample size for the planned analyses. Following methodological guidance, a recommendation was made to increase the sample, and additional data collection was conducted. The study aimed for a minimum of 250 respondents to meet the requirements of correlation analysis and to ensure sufficient statistical power. Upon data cleaning, including checks for outliers and missing values, 208 completed responses were deemed valid. Although the final number of participants fell slightly below the target, the sample size remains acceptable for non-parametric methods, which are appropriate for smaller samples and non-normal data distributions.

### Sample size determination

To test if the sample size of 208 is appropriate, an *a priori* power analysis was conducted in G-Power. The test family was set to F tests, and the statistical test selected was ANOVA: Fixed effects, one way – which aligns with the planned comparison of EI and CB scores across multiple categories. The type of power analysis was set to *a priori – compute sample size, given a, power, effect size*. The significance level was set at 0.5, statistical power (1- *β* error probability) was set at 0.80 to reduce the likelihood of a type II errors and the number of groups was set to five according to the sports categories in the data set. The effect size f was set at 0.25 which is the default medium effect size in G Power for this test. The results indicated a non-centrality parameter (*λ*) of 12.5000000, a critical *F* value of 2.4179625, numerator degrees of freedom of 4 and denominator degrees of freedom of 195. The computed sample size required was 200 participants, with an actual projected power of 0.8097710. Since the participants of the current study is 208, it means and exceeds the recommended sample size planned for analyses, supporting adequate power and improving interpretability of statistical results. Please refer to Table 1.

**Table 1:** Sampling determination computation.

Parameters	Result
Noncentrality parameter *(λ)*	12.5000000
Critical F	2.4179625
Numerator df	4
Denominator df	195
Total sample size	200
Actual power	0.8097710

Source: authors.

### Instruments used

Two standardized instruments and a brief demographic questionnaire were used in this study to measure emotional intelligence, conflict behavior and relevant participant characteristics.

### Wong and Law Emotional Intelligence Scale (WLEIS)

The WLEIS, developed by Wong and Law (2002), was used to assess the EI of Filipino athletes. It is a widely validated self-report instrument consisting of 16 items that measure four dimensions of emotional intelligence: self-emotion appraisal, others’ emotion appraisal, use of emotion, and regulation of emotion. Responses are rated on a 7-point Likert scale ranging from strongly disagree (1) to strongly agree (7). Example of items are “I have a good sense of why I have certain feelings most of the time”, “I am a good observer of the emotions of the people around me”, “I always set goals for myself and then try my best to achieve them”, and I am quite capable of controlling my own emotions.” It was selected because of its high internal consistency and construct validity. Previous studies have reported Cronbach’s alpha values above *a = 0*.85 (Wong and Law, 2002; Wong et al., 2004). To confirm, we also conducted Cronbach’s alpha on our data and results showed *a = 0*.891 indicating high internal consistency and construct validity. Please refer to Table 2.

**Table 2:** Reliability statistics of WLEIS.

Reliability statistics		
Cronbach’s alpha	Cronbach’s alpha based on standardized items	Emotional intelligence N of items
0.887	0.891	16

Source: authors.

### Dutch Test for Conflict Handling (DUTCH)

The DUTCH test, developed by De Dreu et al. (2001), measures five conflict styles: yielding, problem solving, avoiding, forcing, and compromising. The instrument uses scenario-based items to capture how individuals typically respond to conflict situations. It is a 20-item instrument rated on a five point Likert scale from 1(almost never) to 5 (almost always). It examines behavioral conflict styles that represent different degrees of concern for self and concern for others. Example of items are the following: “I give in to the wishes of the other party”, “I do everything to win”, and “I try to realize a middle-of-the-road solution*”*. Reliability coefficients in prior studies have ranged from *a =* 0.70 to 0.86 across subscales. For this study, we calculated Cronbach’s alpha on our data, yielding *a = 0.*952 indicating high internal consistency and construct validity (Table 3).

**Table 3:** Reliability statistics of DUTCH.

Reliability statistics		
Cronbach’s alpha	Cronbach’s alpha based on standardized items	N of items
0.951	0.952	20

Source: authors.

In line with this, brief demographic section in the questionnaire was administered to collect information. In compliance with the ethical standards of the university’s Research and Ethics Board, no personal identifiable information was gathered. Only variables directly relevant to the study’s objectives were included. Since the analysis focused on differences by sex, type of sport, and athletic experience, the questionnaire was limited to these essential demographic indicators.

To address the possibility of common method bias (CMB), several procedure and statistical measure were implemented. First the questionnaire was designed to minimize respondent fatigue and ambiguity. Items from different constructs were mixed to prevent patterned responding and straight lining. Participants were also assured of confidentiality and anonymity to reduce social desirability bias. To further verify whether CMB was present, Harman’s single factor test was done. The analysis showed that the first unrotated factor yielded 3.439% of the total variance. This means that it is below commonly accepted threshold of 50%. This also indicates that CMB is not likely to pose a significant threat to the validity of the results. Please refer to Table 4.

**Table 4:** Common method bias test.

Factor	Initial eigenvalues			Extraction sums of squared loadings		
	% of variance	Cumulative %	Total	% of variance	Cumulative %	Total
1	12.918	35.883	35.883	12.398	34.439	34.439
2	5.405	15.015	50.898			
3	4.284	11.9	62.798			
4	2.795	7.764	70.562			
5	2.059	5.72	76.282			
6	1.375	3.82	80.102			
7	1.269	3.525	83.627			
8	1.218	3.383	87.01			
9	0.643	1.786	88.796			
10	0.594	1.651	90.447			

Source: authors.

### Data collection

Prior to data collection, permission was sought from relevant institutions and sports organizations. Athletes were contacted through coaches, sports clubs, and personal referrals. Data collection was conducted using two modes:

1 Online surveys administered via a secure Google Forms link, circulated through email platforms.
2 Face-to-face survey sessions conducted after training sessions or competitions, where the researcher distributed printed questionnaires. This mode was also use to increase the number of collected data.

The researchers opted for these methodologies to maximize participant engagement and accommodate the diverse schedules and preferences of athletes, thereby ensuring thorough data collection while minimizing any interference with their training and competition regimens.

Respondents were first provided with an informed consent form that explained the purpose of the study, confidentiality provisions, and voluntary participation. Completion of the survey required approximately 20–25 min.

### Statistical treatment

Data were encoded and analysed using SPSS (Statistical Package for the Social Sciences). Consistent with the exploratory, cross sectional quantitative design, the study applied a series of descriptive and inferential statistical procedures to examine patterns within groups. The level of significance was set at *p* < 0.05.

Likewise, it is important to highlight that this study does not aim to develop a new instrument or conduct a psychometric validation. Rather, the objective is to explore patterns of emotional intelligence and conflict management across groups. To date, such group based analysis has not been examined in the context of sports in the Philippines, particularly in relation to our variables of study.

In addition, to clarify, when it comes to the choice of statistical treatment, our decision was based on three intersecting considerations. First, the primary outcome variables, specifically EI scores from the WLEIS and conflict behavior scores from the DUTCH are all measured on Likert scales. Although Likert responses are sometimes treated as interval-level-data, the psychometric literature recommends treating them as ordinal when the distributional assumptions of parametric tests cannot be confirmed, particularly in smaller or moderately sized samples (Liddell and Kruschke, 2018; Bürkner and Vuorre, 2019). As noted by Vrbin (2022) and Weaver et al. (2017), the choice of statistical test depends on several key assumptions. These include whether the data follow a normal distribution, whether the sample variances are equal, and whether the variables are measured on an interval or ratio scale. When all three assumptions are satisfied, parametric tests are appropriate. However, if one or more of these conditions are not met, non-parametric tests are more suitable.

In the present study, the data were not normally distributed, the variances were unequal, and the variables were not measured on an interval or ratio scale. For these reasons, non parametric tests were employed.

With this in mind, the following statistical procedures were performed:

- Descriptive statistics (mean, mode, median, standard deviation, frequency, percentage) to summarise demographic characteristics and levels of emotional intelligence and conflict management styles.
- To examine differences between two groups notably sex and category (individual or team sports), Mann–Whitney U test was applied to examine these. This test is appropriate when the assumptions of parametric tests are not met. EI and CB scores are measured on ordinal Likert scales which do not satisfy interval-level measurement and with small or unequal group sizes, Mann–Whitney is suitable as it detects differences in median scores between two independent groups.
- To determine and compare the types of sports, Jonckheere- Terpstra test was used because an ordered relationship among groups was expected. This test appropriate when independent carriable and dependent variable are ordinal and not normally distributed. Because the data do not meet parametric assumptions and the sample size is small, this is a suitable test for ordered differences among multiple groups.

It is also important to clarify that these tests were used in an exploratory mode. No directional hypotheses specifying which group would score higher were formulated in advance for most comparisons. The tests were employed to determine whether statistically meaningful differences existed across groups, with findings interpreted as preliminary patterns warranting further investigation rather than as confirmatory evidence of theoretical propositions.

### Translation of and scripting the questionnaire

Translation and scripting procedures were considered to ensure that the instruments were appropriate for use with Filipino university athletes. Since the original instruments were written in English and English is widely used as an instructional and communicative language in Philippine universities and sports programmes, no translation into Filipino or other local languages was required. The constructs measured by the WLEIS and DUTCH are also commonly taught and discussed in English in academic settings, making the original language suitable for the target population.

Although translation was not necessary, scripting procedures were implemented to standardise the administration of the survey. Instructions for each instrument were rewritten in simple and clear English to avoid confusion and ensure uniform understanding among participants. Introductory statements were included to explain the purpose of each section, the response scale, and the expected manner of responding. Scripting also ensured that transitions between sections were smooth and that athletes were guided consistently through the online and face to face formats. These steps helped maintain conceptual fidelity to the original instruments while supporting accurate and reliable responses from the participants.

### Ethical consideration

Ethical standards were observed throughout the conduct of this study. Participation was voluntary, and no incentives were provided. Athletes were assured that their responses would remain confidential and would be used solely for academic purposes. Pseudonyms and coded identifiers were used to maintain anonymity. Completed surveys were stored securely in password-protected files and locked storage for physical copies. Ethical clearance was obtained from the university’s Institutional Review Board (IRB) before data collection commenced.

## Results and discussion

### Summary

Before presenting the detailed findings, it is important to situate the contributions of this study within the existing literature. To our knowledge, no prior published study has simultaneously examined emotional intelligence and conflict behavior among Filipino university athletes using culturally grounded framework. Most existing research on EI and conflict management in sports has been conducted in Western or non-Filipino contexts, and studies that have examined athletes have largely focused on performance outcomes without attending to the psychological and cultural mechanisms that shape emotional and interpersonal functioning. This study offers four original contributions. First, it provides a baseline empirical dataset on EI and conflict behavior among Filipino university athletes, a population that has received limited attention in the quantitative sports psychology literature. Second, it demonstrates that emotional regulation, specifically the ROE facet of EI, is sensitive to both sex and sport type, adding to a limited but growing body of evidence suggesting that not all EI facets develop or manifest uniformly across contexts. Third, the absence of significant differences in conflict behavior across sport type and sport category is itself a substantive finding, as it challenges the assumption that sport context is a primary driver of interpersonal behavioral tendencies, and points instead to cultural and individual-level factors as more dominant influences. Fourth, this study is among the first to apply the Filipino Psychology framework of Enriquez (1976, 1977, 1978) specifically the *insider-outsider* distinction of *kapwa*, as an interpretive lens for understanding EI and conflict behavior in a Philippine sports context, offering a culturally responsive theoretical contribution that extends beyond the findings of any single study.

### Socio-demographic profile of participants

Following the survey period, 208 valid responses were obtained. The decision to proceed with non-parametric tests was made as well as acknowledging that although the response rate was lower than anticipated, these methods offer enough statistical power for hypothesis testing, particularly with smaller sample sizes, ordinal data, and potential non-normal distributions.

Table 5 indicates the demographic characteristics of the respondents (*N* = 208). A majority of the participants were male 108 (51.1%) while 100 (48.9%) were female. In terms of academic year level, 68 (32.7%) were first-year students, 29 (13.9%) were in their second year, 46 (22.1%) were in their third year, 46 (22.1%) were in their fourth year, and 19 (9.1%) were classified as irregular status.

**Table 5:** Demographics by gender, year level and sports affiliation.

Variable	Categories/scale	N	Percentage
Sex	Male	108	51.1%
	Female	100	48.9%
Age	18–19	60	28.8%
	20–21	56	26.9%
	22–23	55	26.4%
	24–26	37	17.8%
Year level	First year	68	32.7%
	Second Year	29	13.9%
	Third year	46	22.1%
	Fourth year	46	22.1%
	Irregular	19	9.1%
Sports and its categories			
Individual	Swimming	25	12.02%
	Chess	16	7.69%
	Table Tennis	5	2.40%
	Badminton	9	4.33%
Team	Drum Squad/Pep Squad	55	26.44%
	Basketball	53	25.48%
	Volleyball	16	7.69%
	Table Tennis	14	6.73%
	Badminton	15	7.21%

Source: authors.

The distribution of athletes across sports categories reflected substantial variation among individual and team-based activities. Among individual sports, swimming had the highest representation with 25 athletes (12.02%), followed by chess with 16 athletes (7.69%). Table tennis and badminton were less represented in the individual categories, accounting for five athletes (2.40%) and nine athletes (4.33%) respectively. Furthermore, team sports constituted majority of the sample. The drum or pep squad had the largest number of participants with 55 athletes (26.44%), closely followed by basketball with 53 athletes (25.48%). Volleyball accounted for 16 athletes (7.69%) while table tennis and badminton teams contributed 14 athletes (6.73%) and 15 athletes (7.21%) respectively.

### Emotional intelligence levels of athletes

In terms of perceptions of their respective EI, Table 6 describes the central tendency measures for four dimensions of emotional intelligence among athletes including mean (M), median (Md), mode (Mo) for each item within the facets. Across all dimensions, the median and mode values consistently fall at 6.0. This indicates that most athletes reported high levels of agreement with the statements describing their emotional intelligence.

**Table 6:** Emotional intelligence scores.

Emotional intelligence dimensions	Mean	Median	Mode
Self-emotion appraisal			
I have a good sense of why I have certain feelings most of the time.	5.78	6.0	6
I have good understanding of my own emotions.	5.59	6.0	6
I really understand what I feel.	5.76	6.0	6
I always know whether or not I am happy.	5.69	6.0	6
Other’s emotion appraisal			
I always know others’ emotions from their behavior.	5.52	6.0	6
I am a good observer of others’ emotions.	5.91	6.0	6
I am sensitive to the feelings and emotions of others’ around me.	5.55	6.0	6
I have good understanding of the emotions of others’ around me.	5.90	6.0	6
Use of emotion			
I always set goals for myself and then try my best to achieve them.	6.37	6.0	6
I always tell myself I am a competent person.	5.58	6.0	6
I am a self-motivating person.	5.77	6.0	6
I would always encourage myself to try my best.	6.32	6.0	6
Regulation of emotion			
I am able to control my temper so that I can handle difficulties rationally.	6.09	6.0	6
I am quite capable of controlling my own emotions.	6.26	6.0	6
I can always calm down quickly when I am very angry.	5.88	6.0	6
I have good control of my own emotions.	5.85	6.0	6

Source: authors.

To dive deeper, for SEA, the mean scores ranged from 5.59 to 5.78. This my mean that athletes generally perceive themselves as capable of understanding their own emotions. All items in this dimensions have median and mode of 6.0 and reflects strong self-awareness among respondents. In the dimension of OEA, the mean values ranged from 5.52 to 5.91. This indicates that athletes also report high sensitivity to the emotions of others. Similar to self-appraisal items, all medians and modes are 6.0 and these suggest consistency toward strong interpersonal emotional awareness. For the UOE, the mean scores ranged from 5.58 to 6.37. The highest mean score in the entire table appears to be in this dimension with the item “I always set goals for myself and then try my best to achieve them” (M = 6.37). All median and mode of 6.0 and shows a uniform pattern of high self-reported emotional utilization. Furthermore, the ROE items show mean scores ranging from 5.85 to 6.26 and these indicate high scores of emotional regulation abilities. All items have a median and mode of 6.0. This demonstrates that athletes perceive themselves as capable of managing their emotions in a controlled manner.

Results revealed that EI scores of participants are high. This result affirms the studies of researchers who found that athletes generally exhibit elevated emotional intelligence, particularly in areas like self-awareness and empathy (Öztürk Çelik, 2025).

However, since this is a cross-sectional study and self-reports, it is not possible to determine if these high EI levels are stable over time or subject to change. In addition, the measurement of EI should be substantiated by a different design to corroborate the findings. Future research could employ longitudinal studies or multi-modal assessments, integrating physiological measures or behavioral observations alongside self-report questionnaires, to provide a more comprehensive and robust evaluation of emotional intelligence in athletes (Lobo et al., 2022).

### Conflict behavior scores of athletes

Table 7 presents the central tendency (mean, median, mode) for each item measuring the five conflict behavior styles notably yielding, compromising, forcing, problem solving, and avoiding. Overall, the results show moderate levels of engagement across the five styles, with the variation in the extent to which athletes report using forcing, compromising, and problem solving and less avoiding,

**Table 7:** Conflict behavior scores of athletes.

Conflict behavior	Mean	Median	Mode
Yielding			
I give in to the wishes of the other party	3.40	3.0	3
I concur with the other party	3.43	3.0	3
I try to accommodate the other party	3.79	4.0	4
I adapt to the other parties’ goal and interests	3.58	4.0	4
Compromising			
I try to realise a middle-of-the-road solution	4.02	4.0	3
I emphasize that we have to find a compromise solution	3.87	4.0	5
I insist we both give in a little	3.79	4.0	4
I strive whenever possible towards a fifty-fifty compromise	3.34	4.0	4
Forcing			
I push my own point of view	3.34	3.0	3
I search for gains	3.72	4.0	4
I fight for a good outcome for myself	3.98	4.0	4
I do everything to win	4.27	5.0	5
Problem solving			
I examine issues until I find a solution that really satisfies me and the other party	4.07	4.0	4
I stand for my own and other’s goals and interests	3.85	4.0	3
I examine ideas from both sides to find a mutually optimal solution	3.96	4.0	4
I work out a solution that serves my own as well as other’s interests as good as possible	3.93	4.0	3
Avoiding			
I avoid confrontation about our differences	3.45	3.0	3
I avoid differences of opinion as much as possible	3.25	3.0	3
I try to make differences loom less severe	3.42	3.0	3
I try to avoid a confrontation with the other	3.78	4.0	3

Source: authors.

For Yielding, mean scores range from 3.40 to 3.79, indicating that athletes sometimes accommodate others’ preferences, though this style is not dominant. Items such as “I give in to the wishes of the other party” (M = 3.40, Md = 3.0, Mo = 3) and “I concur with the other party” (M = 3.43, Md = 3.0, Mo = 3) reflect modest agreement, whereas more relational items such as “I try to accommodate the other party” and “I adapt to the other parties’ goal and interests” show slightly higher medians of 4.0, suggesting a tendency toward cooperative behavior when group harmony is at stake.

In the Compromising style, mean scores range from 3.34 to 4.02. This indicates that athletes frequently use middle-ground strategies when faced with conflict. The item “I try to realise a middle-of-the-road solution” has the highest mean in this category (M = 4.02, Md = 4.0), a preference for mutually acceptable outcomes. Notably, “I emphasize that we have to find a compromise solution” shows a mode of 5, suggesting that a subset of athletes strongly endorse compromise-oriented responses.

The Forcing dimension shows a wider spread, with means from 3.34 to 4.27. Items such as “I search for gains” (M = 3.72, Md = 4.0) and “I fight for a good outcome for myself” (M = 3.98, Md = 4.0) indicate moderate self-assertion. The strongest endorsement occurs in the item “I do everything to win” (M = 4.27, Md = 5.0, Mo = 5). This suggests that competitive drive and self-oriented strategies are more strongly endorsed in high-stakes or performance-related conflicts.

For Problem Solving, the mean scores range from 3.85 to 4.07, indicating frequent use of cooperative and analytical strategies. Items such as “I examine issues until I find a solution that really satisfies me and the other party” (M = 4.07, Md = 4.0, Mo = 4) and “I examine ideas from both sides to find a mutually optimal solution” (M = 3.96, Md = 4.0, Mo = 4) suggest that athletes often engage in constructive negotiation and seek solutions that benefit both sides. This reflects a tendency toward collaborative conflict resolution.

In the Avoiding category, mean scores range from 3.25 to 3.78. This may suggest that avoidance strategies are used occasionally but not as strongly as compromising or problem solving. Items such as “I avoid confrontation about our differences” (M = 3.45, Md = 3.0, Mo = 3) and “I avoid differences of opinion as much as possible” (M = 3.25, Md = 3.0, Mo = 3) show moderate levels of agreement. The item “I try to avoid a confrontation with the other” has the highest mean in this category (M = 3.78, Md = 4.0), indicating that some athletes prefer to preserve harmony by withdrawing from direct conflict. This suggests that compromise, forcing, and problem-solving are the preferred methods of conflict resolution among athletes. And, this affirms the study of Ros-Morente et al. (2022) that athletes often prefer direct and assertive strategies for resolving disagreements rather than deferring to others or sidestepping conflict entirely.

In line with this, yielding and avoiding are less common among athletes. This inclination toward direct engagement aligns with the competitive nature inherent in sports, where individuals are often trained to confront challenges directly rather than circumvent them (Marques et al., 2024). The prevalence of compromising and problem-solving approaches further indicates a desire for mutually beneficial outcomes, even in high-pressure situations. Such findings are consistent with previous research suggesting that emotionally intelligent individuals, particularly in demanding fields like sports, are adept at employing effective conflict resolution strategies (Zhou et al., 2025). Moreover, this preference for integrative and distributive conflict behavior styles over avoidance has been observed in various athletic populations, reflecting a proactive approach to interpersonal challenges that can be crucial for team cohesion and individual performance (Kılıç and Duygulu, 2024). These tendencies demonstrate athletes’ developed emotional competences, allowing them to better adapt to stressful situations and control their own emotions and those of others than avoid these (Hassen et al., 2022).

### Relationship between emotional intelligence of athletes by sex

When comparing EI scores of male and female athletes using Mann–Whitney U test in Table 8, a clear pattern began to emerge. The data revealed that the way athletes understand and manage their emotions is not the same across sex.

**Table 8:** Relationship between EI and sex.

Null hypothesis	Test	Sig.^a,b^	Decision
The distribution of I really understand what I feel. is the same across categories of sex.	Independent-Samples Mann–Whitney U Test	0.017	Reject the null hypothesis.
The distribution of I always know others’ emotions from their behavior. is the same across categories of sex.	Independent-Samples Mann–Whitney U Test	0.019	Reject the null hypothesis.
The distribution of I am quite capable of controlling my own emotions is the same across categories of sex.	Independent-Samples Mann–Whitney U Test	0.025	Reject the null hypothesis.
The distribution of I can always calm down quickly when I am very angry. is the same across categories of sex.	Independent-Samples Mann–Whitney U Test	<0.001	Reject the null hypothesis.
The distribution of I have good control of my own emotions. is the same across categories of sex.	Independent-Samples Mann–Whitney U Test	<0.001	Reject the null hypothesis.

Source: authors.

Beginning with self-understanding, the item “I really understand what I feel” showed a significant difference between male and female athletes (*p* = 0.017). This suggests that the depth of emotional self-awareness varies across genders, with one group reporting a stronger sense of understanding their own feelings.

A similar pattern appears in how athletes interpret the emotions of others. For the item “I always know others’ emotions from their behavior,” the difference across gender groups was again significant (*p* = 0.019). This indicates that the ability to read emotional cues from others is not uniformly experienced, pointing to possible differences in interpersonal sensitivity or the social contexts through which male and female athletes develop these skills.

The most notable differences arise in the domain of emotional regulation. The item “I am quite capable of controlling my own emotions” reached significance (*p* = 0.025), suggesting that the sense of emotional control differs between genders. The following two items showed even stronger distinctions. For “I can always calm down quickly when I am very angry,” the *p*-value was less than 0.001, indicating a highly significant difference. Likewise, the item “I have good control of my own emotions” also produced a *p*-value of less than 0.001. These results may reveal that male and female athletes report substantially different experiences in regulating and managing difficult emotions such as anger or stress.

With this in mind, the differences on how Filipino athletes understand their emotions and others and how they regulate their emotions are not the same across sex. According to researchers, female athletes generally exhibit higher scores in emotional appraisal, while male athletes tend to demonstrate superior emotional control and regulation abilities (Rodríguez-Romo et al., 2021). However, on the contrary, though the samples are non-athletic, in the study of Laborde et al. (2014), men had higher levels of EI than women. In other contexts, the study of Lobo et al. (2022) found out that there is no significant difference between sex and EI because EI is a stable ability and a psychological resource which means when an individual learned and mastered EI facets, these will be instilled in the individual’s cognition. Nonetheless, these observed differences suggest that the way emotional intelligence manifests across sex may not be uniform, and that neurobiological and socio-cultural factors could plausibly contribute to these patterns, though the present cross-sectional design does not permit any determination of the direction or cause of these differences. Future research employing longitudinal or experimental designs would be better positioned to examine how and why these patterns emerge.

### Relationship between emotional intelligence of athletes by types of sports

The analysis also explored whether athlete’s emotional regulation differed across various sport categories using Jonckheere-Terpstra test, and results revealed patterns in the facet of regulation of emotions in Table 9. or the item “I am quite capable of controlling my own emotions,” the test produced a significant result (*p* = 0.015), indicating that emotional control does not follow the same distribution across sport types. This suggests that athletes in certain sports may perceive themselves as more emotionally regulated than others. Because this test assesses ordered differences, the result implies a possible trend in which emotional control increases or decreases in a patterned way across the different sport categories.

**Table 9:** Relationship between EI and type of sports.

Null Hypothesis	Test	Sig.^a,b^	Decision
The distribution of I am quite capable of controlling my own emotions is the same across categories of sports category/event.	Independent-Samples Jonckheere-Terpstra Test for Ordered Alternatives	0.015	Reject the null hypothesis.
The distribution of I can always calm down quickly when I am very angry. is the same across categories of sports category/event.	Independent-Samples Jonckheere-Terpstra Test for Ordered Alternatives	0.009	Reject the null hypothesis.

Source: authors.

A similar and even stronger pattern emerged for the item “I can always calm down quickly when I am very angry,” which also showed a significant difference across sport categories (*p* = 0.009). This finding suggests that the ability to recover quickly from anger varies among athletes depending on the type of sport they participate in. It may be possible that some sports may cultivate fast emotional recovery through constant exposure to pressure and rapid decision-making, while others may involve environments where emotional regulation is challenged differently.

These results highlight that while other emotional intelligence facets remain similar, there is a difference in regulation of emotion across sports categories. According to researchers, the facet of self-emotion and others’ emotion appraisal tend to develop earlier in an individual’s emotional development and remain relatively stable because they are shaped by social experience, schooling and everyday interactions (O’Connor et al., 2019). Whereas, the capacity for emotion regulation typically matures later and is more susceptible to environmental influences and specialized training (Valente and Lourenço, 2020). Regulation of emotion is regarded as performance-facing skill that develops through repeated exposure to conflict, stress and emotionally charged situations (Lobo, 2023). Some sports demand emotional control to maintain precision and focus, such as shooting or archery, which require precise emotional management to maintain peak performance, contrasting with sports where emotional expression might be more integrated into competitive strategies (Rubio et al., 2023; Zhou et al., 2025).

### Relationship between emotional intelligence of athletes by category (individual or team)

When comparing athletes from individual sports and team sports (please refer to Table 10) using Mann–Whitney U test, most aspects of emotional intelligence showed similar distributions across groups. However, the statement “I am sensitive to the feelings and emotions of others” displayed a significant difference between the two categories of athletes (*p* = 0.048), leading to the rejection of the null hypothesis. This means that sensitivity to others’ emotions varies depending on whether an athlete participates in an individual or a group sport.

**Table 10:** Relationship between EI and category (individual and team).

Null Hypothesis	Test	Sig.^a,b^	Decision
The distribution of I am sensitive to the feelings and emotions of others is the same across categories of group/individual.	Independent-Samples Mann–Whitney U Test	0.048	Reject the null hypothesis.

Source: authors.

Importantly, this was the only item within the broader Others’ Emotion Appraisal dimension that showed such a distinction. While other items measuring awareness of others’ emotions remained consistent across sport types, this particular item suggests difference: athletes who regularly interact within group environments may develop heightened emotional attunement due to constant interpersonal exchanges, while athletes in individual sports may not encounter the same level of relational demands.

This results suggest that when it comes to individual or team-based sports, the distribution of sensitivity to the feelings and emotions of others is not the same across group or individual events. According to researchers (Kopp and Jekauc, 2018; Rubio et al., 2023), some studies, athletes in team sports demonstrate higher scores in emotional intelligence dimensions like empathy and social skills due to the constant need for coordination and communication within a group setting, whereas individual sport athletes may prioritize self-regulation and emotional control to manage solitary competitive pressures (Kopp and Jekauc, 2018, 2025; Laborde et al., 2015). For instance, contact sports require understanding and perceiving the emotions of others due to direct physical confrontation, while non-contact sports necessitate greater self-regulation in response to internal and environmental factors (Kopp and Jekauc, 2018). In terms of coordination, the necessity for synchronized movements and strategic interdependence further enhances the development of inter-personal emotional skills among team sport participants than individual events (Crombie et al., 2009). Though this might be the case, it is also possible that in the Filipino context, the Filipino values of harmonious social interaction and shared identify influence the heightened sensitivity to others.

### Relationship between conflict behavior of athletes by sex

The analysis of conflict behavior, using Mann–Whitney U test, revealed several meaningful gender differences, suggesting that male and female athletes approach interpersonal tension in distinct ways. The first significant finding emerged in the yielding dimension. The item “I try to accommodate the other party” showed a highly significant difference across genders (*p* = 0.003), indicating that one gender is more inclined to prioritize harmony and adjust to others during conflict. This suggests that accommodating behaviors, which emphasize relational maintenance, may be more typical of one gender group within the athletic context.

Gender differences also appeared in more assertive conflict behaviors. The item “I fight for a good outcome for myself” reached significance (*p* = 0.020), showing that athletes differ in the extent to which they pursue their own interests during conflict. A similar pattern was seen in “I do everything to win” (*p* = 0.019), a statement reflecting a competitive and self-focused orientation toward conflict. These two items highlight that assertive, outcome-driven strategies are not expressed equally across genders and may be shaped by both social expectations and competitive norms within sport environments.

Avoidance-related behaviors also showed gender variation. The item “I avoid a confrontation about our differences” produced a significant result (*p* = 0.025), indicating that athletes differ in their use of avoidance as a method for managing disagreements. The item “I try to avoid a confrontation with the other” also showed significance (*p* = 0.044) which reinforces that avoidance tendencies are not uniform across male and female athletes. Together, these results suggest that some athletes may rely more on withdrawal or disengagement when faced with conflict, whereas others may be more inclined to address or confront differences.

These results suggest that there may be gender differences in conflict resolution strategies rooted in emotional understanding and conflict behavior styles (Ros-Morente et al., 2022). For instance, women may favor collaborative and compromising approaches while men more may resort to competitive and avoidant styles when faced with interpersonal disagreements (Hernández et al., 2022).

However, within the Filipino context, one possible interpretation is that women may be more consistently socialized toward relational sensitivity and harmony-maintenance, which could be associated with higher accommodation tendencies, though this remains a theoretical proposition rather than a finding established by the present data. Similarly, it is plausible that men may be more often encouraged toward assertiveness and independence in competitive settings, which may be consistent with the observed differences in forcing and avoidance patterns. These interpretations are offered as candidate explanations to guide future research rather than as conclusions supported by the current design, which does not permit causal or socializational inferences (see Table 11).

**Table 11:** Relationship between conflict behavior styles and sex.

Null Hypothesis	Test	Sig.^a,b^	Decision
The distribution of I try to accommodate the other party is the same across categories of sex.	Independent-Samples Mann–Whitney U Test	0.003	Reject the null hypothesis.
The distribution of I fight for a good outcome for myself is the same across categories of sex.	Independent-Samples Mann–Whitney U Test	0.02	Reject the null hypothesis.
The distribution of I do everything to win is the same across categories of sex.	Independent-Samples Mann–Whitney U Test	0.019	Reject the null hypothesis.
The distribution of I avoid a confrontation about our differences is the same across categories of sex.	Independent-Samples Mann–Whitney U Test	0.025	Reject the null hypothesis.
The distribution of I try to avoid a confrontation with others is the same across categories of sex.	Independent-Samples Mann–Whitney U Test	0.044	Reject the null hypothesis.

Source: authors.

### Relationship between conflict behavior of athletes by sports

The analysis of conflict behavior across different sport categories revealed a consistent pattern: none of the items showed statistically significant differences in Table 12. Using the Jonckheere–Terpstra test, which evaluates whether an ordered trend exists across groups, all *p*-values exceeded the 0.05 significance threshold. As a result, the null hypothesis was retained for every conflict behavior item.

**Table 12:** Relationship between conflict behavior and type of sports.

Null hypothesis	Test	Sig.a,b	Decision
The distribution of I give in to the wishes of the other part is the same across categories of sports category/event.	Independent-Samples Jonckheere-Terpstra Test for Ordered Alternatives	0.845	Retain the null hypothesis.
The distribution of I concur with the other party is the same across categories of sports category/event.	Independent-Samples Jonckheere-Terpstra Test for Ordered Alternatives	0.98	Retain the null hypothesis.
The distribution of I try to accommodate the other party is the same across categories of sports category/event.	Independent-Samples Jonckheere-Terpstra Test for Ordered Alternatives	0.432	Retain the null hypothesis.
The distribution of I adapt to the other parties’ goals and interests is the same across categories of sports category/event.	Independent-Samples Jonckheere-Terpstra Test for Ordered Alternatives	0.842	Retain the null hypothesis.
The distribution of I try to realize a middle of the road solution is the same across categories of sports category/event.	Independent-Samples Jonckheere-Terpstra Test for Ordered Alternatives	0.896	Retain the null hypothesis.
The distribution of I emphasize that we have to find a compromise solution is the same across categories of sports category/event.	Independent-Samples Jonckheere-Terpstra Test for Ordered Alternatives	0.51	Retain the null hypothesis.
The distribution of I insist we both give in a little is the same across categories of sports category/event.	Independent-Samples Jonckheere-Terpstra Test for Ordered Alternatives	0.167	Retain the null hypothesis.
The distribution of I strive whenever possible towards a fifty-fifty compromise is the same across categories of sports category/event.	Independent-Samples Jonckheere-Terpstra Test for Ordered Alternatives	0.894	Retain the null hypothesis.
The distribution of I push my own point of view is the same across categories of sports category/event.	Independent-Samples Jonckheere-Terpstra Test for Ordered Alternatives	0.235	Retain the null hypothesis.
The distribution of I search for gains is the same across categories of Sports category/event.	Independent-Samples Jonckheere-Terpstra Test for Ordered Alternatives	0.39	Retain the null hypothesis.
The distribution of I fight for a good outcome for myself is the same across categories of sports category/event.	Independent-Samples Jonckheere-Terpstra Test for Ordered Alternatives	0.394	Retain the null hypothesis.
The distribution of I do everything to win is the same across categories of sports category/event.	Independent-Samples Jonckheere-Terpstra Test for Ordered Alternatives	0.55	Retain the null hypothesis.
The distribution of I examine issues until I find a solution that really satisfies me and the other party is the same across categories of sports category/event.	Independent-Samples Jonckheere-Terpstra Test for Ordered Alternatives	0.465	Retain the null hypothesis.
The distribution of I stand for my own and other’s goals and interests is the same across categories of sports category/event.	Independent-Samples Jonckheere-Terpstra Test for Ordered Alternatives	0.632	Retain the null hypothesis.
The distribution of I examine ideas for both sides to find a mutually optimal solution is the same across categories of sports category/event.	Independent-Samples Jonckheere-Terpstra Test for Ordered Alternatives	0.916	Retain the null hypothesis.
The distribution of I work out a solution that serves my own as well as other’s interests as good as possible is the same across categories of sports category/event.	Independent-Samples Jonckheere-Terpstra Test for Ordered Alternatives	0.365	Retain the null hypothesis.
The distribution of I avoid a confrontation about our differences is the same across categories of sports category/event.	Independent-Samples Jonckheere-Terpstra Test for Ordered Alternatives	0.671	Retain the null hypothesis.
The distribution of I avoid differences of opinion as much as possible is the same across categories of sports category/event.	Independent-Samples Jonckheere-Terpstra Test for Ordered Alternatives	0.376	Retain the null hypothesis.
The distribution of I try to avoid a confrontation with the other is the same across categories of sports category/event.	Independent-Samples Jonckheere-Terpstra Test for Ordered Alternatives	0.523	Retain the null hypothesis.
The distribution of I work out a solution that serves well as other’s interests as good as possible is the same across categories of sports category/event.	Independent-Samples Jonckheere-Terpstra Test for Ordered Alternatives	0.274	Retain the null hypothesis.

Source: authors.

This result may suggest that conflict behavior styles are not strongly shaped by type of sports. One possible interpretation of this consistent non-significance is that conflict behavior among Filipino athletes may be more strongly shaped by shared cultural orientations than by sport-specific contexts. Values such as relational harmony, deference to authority figures such as coaches, and team-oriented expectations are broadly embedded in Filipino sports culture regardless of sport type, and these shared orientations could plausibly produce similar conflict behavior patterns across groups.

### Relationship between conflict behavior of athletes by category (individual and teams)

In Table 13, when comparing athletes from individual sports and team sports, the analysis revealed a consistent pattern across all conflict behavior items: none of the behaviors differed significantly between the two groups. Using the Mann–Whitney U test, all p-values exceeded the 0.05 threshold, leading to the retention of the null hypothesis for every item. One possible explanation for this consistency is that conflict behavior of Filipino athletes may be shaped by cultural norms regardless when they are participating in individual or team-based events. Even athletes are embedded within training groups, coaching relationships, and institutional environments, they rely on interpersonal harmony and group cohesion not only for performance but also for social acceptance within the community.

**Table 13:** Relationship between conflict behavior and categories (individual and teams).

Null hypothesis	Test	Sig.a,b	Decision
The distribution of I give in to the wishes of the other part is the same across categories of group/individual.	Independent-Samples Mann–Whitney U Test	0.777	Retain the null hypothesis.
The distribution of I concur with the other party is the same across categories of group/individual.	Independent-Samples Mann–Whitney U Test	0.593	Retain the null hypothesis.
The distribution of I try to accommodate the other party is the same across categories of group/individual.	Independent-Samples Mann–Whitney U Test	0.557	Retain the null hypothesis.
The distribution of I adapt to the other parties’ goals and interests is the same across categories of group/individual.	Independent-Samples Mann–Whitney U Test	0.335	Retain the null hypothesis.
The distribution of I try to realize a middle of the road solution is the same across categories of group/individual.	Independent-Samples Mann–Whitney U Test	0.432	Retain the null hypothesis.
The distribution of I emphasize that we have to find a compromise solution is the same across categories of group/individual.	Independent-Samples Mann–Whitney U Test	0.834	Retain the null hypothesis.
The distribution of I insist we both give in a little is the same across categories of group/individual.	Independent-Samples Mann–Whitney U Test	0.697	Retain the null hypothesis.
The distribution of I strive whenever possible towards a fifty-fifty compromise is the same across categories of group/individual.	Independent-Samples Mann–Whitney U Test	0.51	Retain the null hypothesis.
The distribution of I push my own point of view is the same across categories of group/individual.	Independent-Samples Mann–Whitney U Test	0.179	Retain the null hypothesis.
The distribution of I search for gains is the same across categories of group/individual.	Independent-Samples Mann–Whitney U Test	0.11	Retain the null hypothesis.
The distribution of I fight for a good outcome for myself is the same across categories of group/individual.	Independent-Samples Mann–Whitney U Test	0.666	Retain the null hypothesis.
The distribution of I do everything to win is the same across categories of Group/Individual.	Independent-Samples Mann–Whitney U Test	0.67	Retain the null hypothesis.
The distribution of I examine issues until I find a solution that really satisfies me and the other party is the same across categories of Group/Individual.	Independent-Samples Mann–Whitney U Test	0.869	Retain the null hypothesis.
The distribution of I stand for my own and other’s goals and interests is the same across categories of Group/Individual.	Independent-Samples Mann–Whitney U Test	0.288	Retain the null hypothesis.
The distribution of I examine ideas for both sides to find a mutually optimal solution is the same across categories of Group/Individual.	Independent-Samples Mann–Whitney U Test	0.666	Retain the null hypothesis.
The distribution of I work out a solution that serves my own as well as other’s interests as good as possible is the same across categories of Group/Individual.	Independent-Samples Mann–Whitney U Test	0.318	Retain the null hypothesis.
The distribution of I avoid a confrontation about our differences is the same across categories of Group/Individual.	Independent-Samples Mann–Whitney U Test	0.297	Retain the null hypothesis.
The distribution of I avoid differences of opinion as much as possible is the same across categories of Group/Individual.	Independent-Samples Mann–Whitney U Test	0.225	Retain the null hypothesis.
The distribution of I try to avoid a confrontation with the other is the same across categories of Group/Individual.	Independent-Samples Mann–Whitney U Test	0.092	Retain the null hypothesis.
The distribution of I work out a solution that serves well as other’s interests as good as possible is the same across categories of Group/Individual.	Independent-Samples Mann–Whitney U Test	0.452	Retain the null hypothesis.

Source: authors.

### Understanding EI and conflict behavior through Filipino Psychology

**Figure 4:**
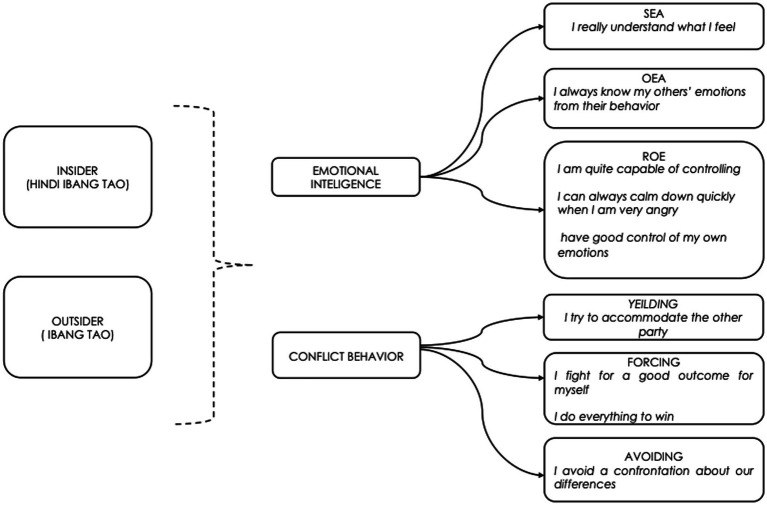
Mapping the connection among Filipino Psychology, EI, and conflict behavior constructs.

Since we already mapped the relationships of EI and CB with athlete’s demographic antecedents, Figure 4 illustrates how significant dimensions and facets of emotional intelligence and conflict behavior among Filipino athletes can be understand through Filipino Psychology framework (insider and outsider perspectives). And for the sake of clarity and depth, we explore interplay of these concepts to better explain why certain facet and specific conflict behavior are significant to the Philippine settings.

With this in mind, the left side in the figure represents the cultural distinction of insider and outsider relationships, which shapes how individuals interpret, understand and regulate emotions. The right side presents the specific facets (SEA, OEA, ROE) and conflict behavior styles (yielding, forcing, avoiding) examined in the study. It also highlights that EI processes such as SEA, OEA, and ROE (except UEO) are influenced by interactions that occur with insiders or outsiders. Filipinos tend to share emotions more openly with insiders and exercise restraints with outsiders. Similarly, conflict behavior styles shown on the right side can be also interpreted through this cultural lens. Yielding behaviors align with maintaining social harmony especially in insider groups and less with outsiders. Forcing may appear more apparent in outsider contexts where assertiveness is more acceptable because maintaining good relationships is weaker. Avoiding behaviors, on the other hand, is associated with avoiding tensions and preserving social order because of values of Filipino harmony. The distinction between individualistic and collectivistic cultures further elucidates these behavioral nuances, as collectivistic societies, like the Philippines, often prioritize group cohesion and relational harmony over individual assertive expression, particularly in emotionally charged situations (Ruiz and Yabut, 2024).

Although we acknowledge that the specific cultural context of Filipino athletes influences these dynamics, universal principles of emotional intelligence in sports may also be applicable. The generalizability of findings from single cultural and sport-specific contexts like the Philippines should be approached with caution and it needs further research across diverse athletic and national settings as noted by Nam et al. (2025).

To understand more clearly, we expand the discussion below:

Emotional intelligence and Filipino Psychology.

In Filipino Psychology, as mentioned above, social interactions are strongly shaped by distinction between insiders (hindi ibang tao) and outsiders (ibang tao) (Ruiz and Yabut, 2024). This dichotomy, rooted in the concept of *kapwa*, profoundly influences how individuals perceive and manage emotional expressions and interpersonal conflicts, particularly in high-stakes environments like competitive sports. It is crucial for understanding how emotional intelligence manifests in Filipino athletes, impacting their leadership dynamics, conflict resolution strategies, and overall team cohesion of the team. The concept of *kapwa* and it covers both shared identity and the recognition of others, dictates the appropriate display and regulation of emotions, thereby influencing how athletes navigate competitive pressures and interpersonal challenges (Soriano-Vázquez et al., 2023). It influences how individuals use their EI and choose behavior in tense situation.

Self-emotional appraisal and *loob* or the insider self.

SEA reflects the ability to understand ones own feelings (Wong and Law, 2002). In Filipino culture, EI awareness is often tied to *loob* or inner self which is shared more openly with insiders than outsiders. It is theoretically consistent to propose that Filipino athletes may feel more comfortable acknowledging their emotions in insider relationships, where trust and shared identity could encourage greater emotional openness, while exercising more restraint with those perceived as outsiders. This proposition is grounded in the *kapwa* framework and supported by broader cultural psychology literature, but it has not been directly tested in the present study and should be treated as a conceptual interpretation rather than an empirically confirmed pattern.

Others’ emotion appraisal and *pakikiramdam*. Pakikiramdam is a Filipino socio-personal construct signifying shared inner perception, or “feeling for another” (Cheng and Liu, 2024, p. 16). It is a Filipino value that underpins the accurate recognition and interpretation of others’ emotional states, a core component of Others’ Emotion Appraisal. This capacity for empathetic understanding is particularly vital in team sports, where athletes must anticipate and respond to teammates’ emotional cues to foster cohesion and coordinated action (Kopp and Jekauc, 2018). This intricate understanding allows athletes to adjust their communication and behavior, thereby mitigating potential conflicts and strengthening interpersonal bonds within the team (Kopp and Jekauc, 2018). This skill is stronger within the insider relationships where athletes feel responsible for maintaining harmony. However, when the individual is perceived as an outsider, the motivation to understand emotions of others may be weaker or responses may be more guarded.

Regulation of emotion and *hiya*-based emotional control.

Regulating emotions is deeply shaped by cultural expectations of restraint and social dignity in Filipino culture. Specifically, *hiya* (modesty) plays a significant role in dictating emotional expression, often leading athletes to suppress outward displays of strong emotions to preserve social harmony and avoid causing discomfort to others. *Hiya* encourages Filipinos to manage anger, frustrations or strong emotions especially in the presence of outsiders to avoid embarrassment or tense situation. Filipino athletes may regulate emotions more intensely to outsiders or not part of their team while expressing true emotions and frustrations with trusted insiders, such as close teammates or coaches (Behm and Carter, 2021; Kopp and Jekauc, 2018). This cultural framing offers one possible conceptual explanation for why ROE emerged as sensitive to both sport type and sex in the present data. The expectation of emotional restraint in certain social contexts may interact with gender-related socialization norms in ways that shape how emotional regulation is practiced and perceived differently across groups. However, this remains an interpretive proposition: the data show that differences in ROE exist across sex and sport type, but they do not reveal the mechanisms responsible for those differences. Establishing whether hiya and insider-outsider dynamics genuinely mediate these patterns would require designs capable of measuring these cultural constructs directly.

Conflict behavior and Filipino Psychology.

In Filipino culture, conflict behavior is heavily influenced by indirect communication styles and a strong emphasis on maintaining smooth interpersonal relationships wherein harmony over direct confrontation is prioritized (Cheng and Liu, 2024; Kopp and Jekauc, 2018). This cultural inclination often leads to indirect strategies for conflict resolution, such as *pakikisama* (getting along with others) and *pakikipagkapwa-tao* (humanity towards others) to prevent open discord and preserve social cohesion (Behm and Carter, 2021).

Yielding behavior and *pakikisama* and *pakikipagkapwa-tao*.

Yielding behavior, for instance, maintain smooth relationship within insider groups especially when it requires *pakikisama and pakikipagkapwa-tao*. Filipino athletes may yield more readily to teammates or coaches considered insiders to preserve unity and harmony. With outsiders, yielding may be less frequent because the relational obligation is weaker. This also helps explain gender or interpersonal differences in yielding. Conversely, it is theoretically plausible that more assertive or contending behavior could emerge in interactions with outsiders, particularly when the protection of ingroup honor or interests is perceived to be at stake. This proposition is consistent with the logic of the *kapwa* framework, but it extends beyond what the present data can confirm, as the study did not measure insider-outsider dynamics directly or observe behavior in specific relational contexts.

Forcing behavior and asserting *loob*.

Forcing behavior such as fighting for one’s desired outcome are more acceptable in outsider relationship where the risk of disrupting harmony is lower. Filipino athletes may assert themselves more when interacting with those perceived as outsiders or when competitiveness overrides relational obligations. This assertiveness can be a strategic display, aiming to influence opponents or establish dominance, particularly as athletes may deliberately employ expressive behaviors to gain a psychological advantage within a competition (Kopp et al., 2021). Such deliberate nonverbal expressions after critical moments in a match can be observed in athletes, where their emotional displays are not merely reactive but strategically employed to communicate intent or influence opponents (Adams et al., 2025). Furthermore, sex dimension also influences how forcing is practiced. This aligns with research indicating that individuals with a dominant interdependent self-construal may prioritize their own image or position, potentially leading to more assertive conflict management strategies in specific contexts (Yamini et al., 2023). And, this is prevalent when Filipino athletes assert dominance more when the relationship does not demand *pakikisama* (getting along with others).

Avoiding behavior and *pag-iwas ng gulo*.

In Filipino culture, avoidance is strongly tied to hiya and cultural value of pag-iwas ng gulo (avoiding trouble). Filipinos often avoid direct confrontation especially when outsiders, to prevent social tension. According to Yamini et al. (2023), *this avoidance behavior is strategically employed to maintain harmony and avert potential embarrassment, both for themselves and others, in situations where direct engagement might escalate conflict* (Yamini et al., 2023, p. 393). Among insiders, avoidance may also emerge to protect relationships from escalation. Although avoidance can sometimes be perceived negatively, however, in the Filipino context, it may often functions as a culturally sanctioned strategy for preserving social equilibrium and preventing overt hostility.

## Conclusion

This study examined emotional intelligence and conflict behavior among 208 Filipino university athletes using an exploratory cross-sectional quantitative design. Rather than testing a relationship between EI and conflict behavior as linked constructs, the study examined three demographic and contextual variables: sex, type of sport, and sport category. The conclusions below are drawn strictly from what the statistical analyses revealed.

With regard to emotional intelligence, athletes reported high levels across all four EI facets, with median and mode scores of 6.0 on the WLEIS across most items, suggesting that this sample perceives itself as emotionally competent. Significant differences by sex emerged primarily in the regulation of emotion facet, with several ROE items showing statistically significant differences between male and female athletes. Differences in the self-emotion appraisal and others’ emotion appraisal facets were also observed for specific items, though not uniformly across all indicators. With regard to sport type, significant differences were found only in the regulation of emotion facet, specifically in the capacity to control emotions and to recover quickly from anger. No significant differences in EI were found across sport category, with the exception of one item within the others’ emotion appraisal dimension, which showed a significant difference between individual and team sport athletes. These findings suggest that among the EI facets examined, emotional regulation is the dimension most sensitive to both sex and sport type in this sample.

With regard to conflict behavior, significant differences by sex were found across specific items within the yielding, forcing, and avoiding dimensions, suggesting that male and female athletes in this sample may differ in how they accommodate others, assert their own interests, and withdraw from confrontation. No significant differences in conflict behavior were found across sport type or sport category, suggesting that in this sample, conflict behavior patterns are not meaningfully differentiated by the sport context in which athletes compete.

Interpreted through the Filipino Psychology framework of Enriquez (1977), particularly the insider-outsider distinction of kapwa, these patterns are theoretically consistent with cultural values such as hiya, pakikisama, and pakikiramdam, which shape emotional expression and interpersonal behavior differently depending on relational proximity. This interpretive lens is offered as a conceptual proposition to guide future research rather than as a conclusion confirmed by the present data, as the Filipino Psychology constructs were not operationalized or statistically tested in this study.

Having said these, this study has several limitations. First, the use of convenience sampling restricts the generalizability of the findings, as the sample reflects mainly university athletes. Second, data were collected through self-report measures, which may be influenced by social desirability and cultural norms such as *hiya* and *pakikisama*. Third, the cross-sectional design prevents causal interpretations of the relationships between emotional intelligence and conflict behavior. Fourth, in this study, we did not statistically test the relationship among Filipino Psychology, EI and CB. We encourage the researcher to explicitly explore the concepts of outsider and insider in line with Filipino Psychology framework or any indigenous framework, using sequential design or qualitative design, that can explain meaningfully how it influence EI and CB. Lastly, the sports categories used in this study were treated broadly without deeper consideration of team climate or sports-specific interpersonal demands that may influence certain patterns. We recommend that in future research, researchers should consider broader and more diverse sampling to improve generalizability. Mixed-methods or multi-informant designs as well as analysis of data collection modes (face to face, online, telephone) may help reduce biases inherent to self-report data. Longitudinal approaches are recommended to capture how emotional and conflict related behaviors develop over time. Studies incorporating Filipino cultural constructs and contextual sports factors may deepen understanding of the findings. Finally, sports psychologists and coaches may develop culturally grounded EI and conflict-management programmes tailored to the needs of the Filipino athletes.

## Data Availability

The raw data supporting the conclusions of this article will be made available by the authors, without undue reservation.

## Footnotes

1 To clarify, conflict management styles refer to strategies of individuals when dealing with conflict (i.e., party in conflict, third party) while conflict behaviors refer to expression or manifestation of the chosen style in real situations (i.e., avoiding, compromising, problem-solving, forcing).

2 It is worthy to highlight that Filipino Atheletes’ Sports Performance is not part of the study but could be explored by future researchers. Our aim to lay the groundwork first before understanding how athletes perform.

## References

[ref1] Adams Y. Augenstein M. Furley P. Krieg A. Born P. Helmich I. (2025). Female athletes explicitly gesture in emotional situations. Front. Psychol. 15:1526542. doi: 10.3389/fpsyg.2024.1526542, 39839917 PMC11747790

[ref2] Assi M. D. Eshah N. F. Rayan A. (2022). The relationship between mindfulness and conflict resolution styles among nurse managers: a cross-sectional study. SAGE Open Nurs. 8:371. doi: 10.1177/23779608221142371, 36467312 PMC9709178

[ref3] Behm D. G. Carter T. B. (2021). Empathetic factors and influences on physical performance: a topical review. Front. Psychol. 12:686262. doi: 10.3389/fpsyg.2021.686262, 34335399 PMC8316856

[ref9003] Boncompagni I Casagrande M (2019) Executive Control of Emotional Conflict. Frontiers in Psychology, 10:359. doi: 10.3389/fpsyg.2019.00359 30873080 PMC6401621

[ref4] Burcea Ș. G. Sabie O. M. (2020). Is emotional intelligence a determinant factor for leader’s skills development? Essential literature perspectives. Manag. Econ. Rev. 5, 68–79. doi: 10.24818/mer/2020.06-06

[ref5] Bürkner P. C. Vuorre M. (2019). Ordinal regression models in psychology: a tutorial. Adv. Methods Pract. Psychol. Sci. 2, 77–101. doi: 10.1177/2515245918823199

[ref6] Cheng P. Liu H. (2024). A structural model of EFL teachers’ physical activity, emotion regulation, and competence for online teaching. BMC Psychol. 12:753. doi: 10.1186/s40359-024-01753-2, 38715133 PMC11077802

[ref7] Corbí R. G. Pozo-Rico T. Sánchez B. S. Costa J. L. C. (2019). Can emotional intelligence be improved? A randomized experimental study of a business-oriented EI training program for senior managers. PLoS One 14:e0224254. doi: 10.1371/journal.pone.0224254 31644585 PMC6808549

[ref8] Crombie D. Lombard C. Noakes T. D. (2009). Emotional intelligence scores predict team sports performance in a national cricket competition. Int. J. Sports Sci. Coach. 4, 209–224. doi: 10.1260/174795409788549544

[ref9] de Dreu C. K. W. Evers A. Beersma B. Kluwer E. S. Nauta A. (2001). A theory-based measure of conflict management strategies in the workplace. J. Organ. Behav. 22, 645–668. doi: 10.1002/job.107

[ref10] Ding C. Ramdas M. Mortillaro M. (2024). Editorial: emotional intelligence in applied settings: approaches to its theoretical model, measurement, and application. Front. Psychol. 15:1387152. doi: 10.3389/fpsyg.2024.1387152, 38515968 PMC10955050

[ref11] Diotaiuti P. Corrado S. Mancone S. Falese L. (2021). Resilience in the endurance runner: the role of self-regulatory modes and basic psychological needs. Front. Psychol. 11:558287. doi: 10.3389/fpsyg.2020.558287, 33488440 PMC7815764

[ref12] Enriquez V. G. (1977). Filipino psychology in the third world. Philipp. J. Psychol. 35, 1–17.

[ref9002] Enriquez V. G. (1978). Kapwa: A core concept in Filipino social psychology. Philippine SocialSciences and Humanities Review, 42.

[ref9001] Enriquez V. G. (1976). Sikolohiyang Pilipino: Perspektibo at direksyon (Filipino psychology:perspective and directioon). In Antonio L. F. Reyes E. S. Pe R. E. Almonte N. R. (Eds.), Ulat ngUnang Pambansang Kumperensya sa Sikolohiyang Pilipino (Proceedings of the First NationalConference on Filipino Psychology) (pp. 221–243). Quezon City: Pambasang Samahan saSikolohiyang Pilipino.

[ref13] Gunkel M. Schlägel C. Engle R. L. (2013). Culture’s influence on emotional intelligence: an empirical study of nine countries. J. Int. Manag. 20, 256–274. doi: 10.1016/j.intman.2013.10.002

[ref14] Hassen S. B. Chelbi I. E. B. Kaabi S. Hamrrouni S. (2022). Impact of emotional competences on Tunisian athletes during the containment period. Adv. Phys. Educ. 12, 78–94. doi: 10.4236/ape.2022.122007

[ref15] Havenga W. Visagie J. (2006). Interpersonal conflict-handling styles used in public and private sector organisations: a comparative study. SA J. Ind. Psychol. 32, 32–41. doi: 10.4102/sajip.v32i1.223

[ref16] Hernández I. L. Gómez R. G. Morales M. T. V. Vélez A. L. L. (2022). Pedagogical contexts when gender and emotions intersect with the body. Int. J. Environ. Res. Public Health 19:15510. doi: 10.3390/ijerph192315510, 36497583 PMC9738121

[ref17] Jenita J. Hasbi M. Prabowo H. Saling S. Idrus S. (2024). The relationship between emotional intelligence and leadership effectiveness. Global Int. J. Innov. Res. 2, 1–1915. doi: 10.59613/global.v2i8.266

[ref18] Kılıç M. Ö. Duygulu S. (2024). Investigating conflict management styles and emotional intelligence of unit charge nurses. Mediterr. Nurs. Midwifery 4, 166–175. doi: 10.4274/mnm.2024.23150

[ref19] Kopp A. Jekauc D. (2018). The influence of emotional intelligence on performance in competitive sports: a meta-analytical investigation. Sports 6:175. doi: 10.3390/sports6040175, 30551649 PMC6316207

[ref20] Kopp A. Jekauc D. (2025). Trait emotional intelligence in competitive sports: differences across sports disciplines. BMC Psychol. 13:563. doi: 10.1186/s40359-025-02563-w 40089773 PMC11909853

[ref21] Kopp A. Reichert M. Jekauc D. (2021). Trait and ability emotional intelligence and its impact on sports performance. Sports 9:60. doi: 10.3390/sports9050060, 34068536 PMC8170878

[ref9006] Laborde S. Dosseville F. Guillén F. Chávez E. (2014). Validity of the trait emotional intelligence questionnaire in sports and its links with performance satisfaction. Psychology of Sport and Exercise, 15, 481–490. doi: 10.1016/j.psychsport.2014.05.001

[ref9007] Laborde S. Guillén F. Dosseville F. Allen M. S. (2015). Chronotype, sport participation, and positive personality-trait-like individual differences. Chronobiology International, 32, 942–951. doi: 10.3109/07420528.2015.1055755 26181469

[ref22] Liddell T. M. Kruschke J. K. (2018). Analyzing ordinal data with metric models: what could possibly go wrong? J. Exp. Soc. Psychol. 79, 328–348. doi: 10.1016/j.jesp.2018.08.009

[ref9008] Liu Y. Zeng B. Chang L. (2025). Examining the links between sense of belonging, conflict resolution skills, emotional intelligence, and life satisfaction in Chinese universities. BMC Psychology, 13. doi: 10.1186/s40359-025-02742-9 PMC12020210 40275378

[ref23] Lobo K. G. (2023). Emotional intelligence and conflict management in family firms: a systematic literature review and bibliometric analysis. DLSU Business Review. 32:14. doi: 10.59588/2243-786X.1166

[ref24] Lobo K. G. Rivera J. P. R. Hayati B. (2022). A non-parametric analysis of emotional intelligence among family businesses in the Philippines. Vision Bus. Perspect. 30:138. doi: 10.1177/09722629221092138

[ref9009] Mancini G Biolcati R Joseph D Trombini E Andrei F (2022) Editorial: Emotional intelligence: Current research and future perspectives on mental health and individual differences. Front. Psychol. 13:1049431. doi: 10.3389/fpsyg.2022.1049431 36312187 PMC9608532

[ref25] Marques R. Violant V. Silva E. D. da. (2024). Emotions and decision-making in boardrooms: a systematic review. Front. Psychol., 15:147. doi:doi: 10.3389/fpsyg.2024.1473175 PMC11603364 39610450

[ref26] Mayer J. D. Caruso D. R. Salovey P. (2016). The ability model of emotional intelligence: principles and updates. Emotion Rev. 8, 290–300. doi: 10.1177/1754073916639667

[ref27] McGinnis E. J. (2018). Developing the emotional intelligence of undergraduate music education majors. J. Music Teacher Educ. 27, 11–22. doi: 10.1177/1057083717723919

[ref28] Messarra L. C. Karkoulian S. El-Kassar A.-N. (2016). Conflict resolution styles and personality. Int. J. Prod. Perform. Manag. 65, 792–810. doi: 10.1108/IJPPM-01-2016-0014

[ref29] Mitić P. Nedeljković J. Takšić V. Sporiš G. Stojiljković N. Milčić L. (2020). Sports performance as a moderator of the relationship between coping strategy and emotional intelligence. Kinesiology 52, 281–291. doi: 10.26582/k.52.2.15

[ref30] Mon-López D. Blanco-García C. Acebes-Sánchez J. Rodríguez-Romo G. Nieto M. M. Martín-Castellanos A. . (2023). Emotional intelligence in Spanish elite athletes. Sports 11:160. doi: 10.3390/sports11080160, 37624140 PMC10458113

[ref31] Murphy L. S. (2009). Developing emotional intelligence as a means to increase team performance. World Rev. Entrep. Manag. Sustain. Dev. 5, 193–203. doi: 10.1504/WREMSD.2009.023762

[ref32] Nam K.-W. Ha J.-H. Yoon S. (2025). Coaching knowledge, sport emotion, and perceived performance in Korean judoka. Front. Psychol. 16:1615383. doi: 10.3389/fpsyg.2025.1615383, 40969478 PMC12442557

[ref33] Nozaki Y. Koyasu M. (2013). Trait emotional intelligence and interaction with ostracized others’ retaliation. PLoS One 8:579. doi: 10.1371/journal.pone.0077579 PMC3806795 24194890

[ref34] O’Boyle E. H. Humphrey R. H. Pollack J. M. Hawver T. H. Story P. A. (2010). The relation between emotional intelligence and job performance: a meta-analysis. J. Organ. Behav. 32, 788–818. doi: 10.1002/job.714

[ref9004] O’Connor P. J. Hill A. Kaya M. Martin B. (2019). The Measurement of Emotional Intelligence: A Critical Review of the Literature and Recommendations for Researchers and Practitioners. Frontiers in Psychology, 10. doi: 10.3389/fpsyg.2019.01116 PMC6546921 31191383

[ref9005] Öztürk Çelik D. (2025). Emotional Intelligence and Psychological Well-Being of Turkish Physical Education and Sports Athlete–Students: The Mediating Roles of Self-Efficacy and Burnout. Behavioral Sciences, 15, 314. doi: 10.3390/bs15030314 40150209 PMC11939540

[ref35] Peris-Delcampo D. Núñez A. Ortiz-Marholz P. Zafra A. O. Cantón E. Ponseti J. . (2024). The bright side of sports: a systematic review on well-being, positive emotions, and performance. BMC Psychol. 12:284. doi: 10.1186/s40359-024-01769-8, 38773650 PMC11106975

[ref36] Rahim M. A. (2002). Toward a theory of managing organizational conflict. Int. J. Confl. Manag. 13, 206–235. doi: 10.1108/eb022874

[ref37] Rodríguez-Romo G. Blanco-García C. Díez-Vega I. Acebes-Sánchez J. (2021). Emotional intelligence of undergraduate athletes. Front. Psychol. 12:609154. doi: 10.3389/fpsyg.2021.609154 33584476 PMC7875876

[ref38] Ros-Morente A. Farré M. Quesada-Pallarès C. Guiu G. F. (2022). Evaluation of happy sport, an emotional education program for assertive conflict resolution. Int. J. Environ. Res. Public Health 19:2596. doi: 10.3390/ijerph19052596, 35270288 PMC8909401

[ref39] Rubio I. M. Ángel N. G. Teixeira A. R. Brito-Costa S. (2023). Relationships between informal sports leadership and emotional intelligence. Sustainability 15:14571. doi: 10.3390/su151914571

[ref40] Ruiz W. D. G. Yabut H. J. (2024). Autonomy and identity: the role of developmental tasks in adolescent well-being. Front. Psychol. 15:1309690. doi: 10.3389/fpsyg.2024.1309690 38659674 PMC11042260

[ref41] Salovey P. Mayer J. D. (1990). Emotional Intelligence. Imagination Cogn. Pers. 9, 185–211. doi: 10.2190/DUGG-P24E-52WK-6CDG (Original work published 1990)

[ref42] Santiago A. Mateo A. (2020). The emotional intelligence of founders and successors. Int. J. Employ. Stud. 28, 82–98. doi: 10.3316/informit.643601478855938

[ref43] Shih H.-A. Susanto E. (2010). Conflict management styles, emotional intelligence, and job performance. Int. J. Confl. Manag. 21, 147–168. doi: 10.1108/10444061011037387

[ref44] Soriano-Vázquez I. Castro M. C. Morales-García W. C. (2023). Emotional intelligence as a predictor of job satisfaction. Front. Public Health 11:1249020. doi: 10.3389/fpubh.2023.1249020 38026285 PMC10667434

[ref9010] Valente S. Lourenço A. A. (2020). La inteligencia emocional marca la diferencia: El impacto de las habilidades de inteligencia emocional del profesorado en las estrategias de manejo de conflictos en el aula. Know and Share Psychology, 1. doi: 10.25115/kasp.v1i4.4249

[ref45] Vrbin C. M. (2022). Parametric or nonparametric statistical tests: considerations when choosing the most appropriate option for your data. Cytopathology 33, 663–667. doi: 10.1111/cyt.13174, 36017662

[ref46] Weaver K. F. Morales V. Dunn S. L. Godde K. Weaver P. F. (2017). “Parametric versus nonparametric tests,” in An Introduction to Statistical Analysis in Research: With Applications in the Biological and life Sciences. 1st ed (New Jersey, USA: John Wiley & Sons), 191–194.

[ref47] Wong C. S. Law K. S. (2002). The effects of leader and follower emotional intelligence on performance and attitude. Leadersh. Q. 13, 243–274. doi: 10.1016/S1048-9843(02)00099-1

[ref480000] Wong C. S. Law K. S. Wong P. -M. (2004). Development and validation of a forced choice emotional intelligence measure for Chinese respondents in Hong Kong. Asia Pacific Journal of Management, 21, 535–559. doi: 10.1023/B:APJM.0000048717.31261.d0

[ref48] Yamini S. Fousiani K. Wisse B. (2023). Self-construal, face concerns, and conflict management strategies: a meta-analysis. Cross Cult. Strateg. Manag. 30, 375–402. doi: 10.1108/CCSM-07-2021-0130

[ref49] Zhou J. Qin S. Jia T. Shen M. Liu H. Tian W. . (2025). The relationship between principals’ emotional intelligence and conflict management. Front. Psychol. 16:1548185. doi: 10.3389/fpsyg.2025.1548185 40242737 PMC12001525

